# Association between the systemic inflammatory response index and acute ischemic stroke in young people

**DOI:** 10.3389/fneur.2025.1644963

**Published:** 2025-11-19

**Authors:** He Wang, Huimin Qiao, Xiangjian Zhang, Peng Wu, Yuxiao Gao, Xin Liu, Haisen An, Wanyue Ge, Meiling Song, Yatong Wang, Ya Wen, Yi Yang

**Affiliations:** 1 Department of Neurology, The Second Hospital of Hebei Medical University, Shijiazhuang, Hebei, China; 2 Key Laboratory of Clinical Neurology, Hebei Medical University, Ministry of Education, Shijiazhuang, Hebei, China; 3 Neurological Laboratory of Hebei Province, Shijiazhuang, Hebei, China; 4 Hebei Key Laboratory of Vascular Homeostasis and Hebei Collaborative Innovation Center for Cardio Cerebrovascular Disease, The Second Hospital of Hebei Medical University, Shijiazhuang, Hebei, China

**Keywords:** inflammation, infarct volume, systemic inflammatory response index, youth acute ischemic stroke, intracranial artery stenosis

## Abstract

**Objective:**

The study aimed to explore the correlation between the systemic inflammatory response index (SIRI) and acute ischemic stroke in youth.

**Methods:**

A retrospective study was conducted. A total of 90 patients aged 18–45 years with acute ischemic stroke were included in the youth cerebral infarction (YCI) group, and 50 patients within the same age bracket without stroke or intracranial atherosclerosis, who were hospitalized during the same period, were included in the control group. Clinical information, blood biochemical indicators, and imaging data of the participants were analyzed. Binary logistic regression was used to assess the independent association between the SIRI and YCI. Furthermore, a subgroup analysis was performed on YCI patients. The subgroup classification included (i) infarct volume grouping; (ii) intracranial artery stenosis grouping; (iii) the Trial of ORG 10172 in Acute Stroke Treatment (TOAST) classification grouping; (iv) infarct distribution grouping; and (v) vasculopathy grouping.

**Results:**

The SIRI values were higher in the YCI group compared to the control group (*p* = 0.005). After adjusting for confounding factors, multivariate logistic regression confirmed that the SIRI is an independent factor associated with YCI (OR = 1.692,95% CI:1.045–2.739, *p* = 0.032). The receiver operating characteristic (ROC) curve showed that the optimal cutoff value for the SIRI as a predictor of YCI was 0.83*10^9/L, with corresponding sensitivity and specificity of 77.8 and 50%, respectively. The AUC was 0.643, with a 95%CI of 0.54–0.74 and a *p*-value of = 0.005. The subgroup analysis results were as follows: (i) There was no statistically significant difference in the SIRI values among the infarct volume groups (*p* = 0.633). (ii) The SIRI values in the severe stenosis group were higher than those in the non-stenosis and mild-to-moderate stenosis groups (*p* < 0.001). Binary logistic regression analysis showed that the SIRI was an independent associated factor for severe stenosis (original OR = 3.346,95% CI = 1.761–6.359, *p* < 0.001; corrected OR = 5.278,95% CI = 2.317–12.022, *p* < 0.001). (iii) The SIRI values in the large-vessel atherothromboembolic (LAA) group were higher than those in the small-vessel disease (SVD) group (*p* = 0.003). (iv) There was no statistically significant difference in the SIRI values between the infarct distribution groups (*p* = 0.572). (v) There was no statistically significant difference in the SIRI values between the vasculopathy groups (*p* = 0.345).

**Conclusion:**

The SIRI is independently associated with YCI and is significantly linked to severe intracranial arterial stenosis and the LAA subtype.

## Introduction

Although the incidence of ischemic stroke increases with age, it affects a substantial proportion of young individuals, accounting for 10–20% of patients aged 18–45 years. Stroke in young adults is particularly concerning due to its association with a high risk of disability, recurrence, and mortality (1–3), posing a significant socioeconomic burden. The etiology of ischemic stroke in this population is more diverse than in older adults (2). While atherosclerosis remains the most common cause (4), other factors such as immune disorders, infections, and related vascular injuries also play significant roles (5). Inflammation serves as a critical pathological basis for atherosclerosis (6), where specific inflammatory biomarkers and underlying inflammatory processes contribute to plaque rupture and arterial thrombotic events (7).

Recently, the systemic inflammatory response index (SIRI) has been proposed as a novel, integrated indicator of systemic inflammation. Calculated from neutrophil, monocyte, and lymphocyte counts, the SIRI is defined by the formula SIRI = neutrophils×monocytes/lymphocytes. It has proven effective in reflecting inflammatory status and predicting the prognosis and risk of various conditions, including sepsis and metabolic dysfunction-associated fatty liver disease (8, 9). Compared to traditional single-parameter inflammatory markers, the SIRI integrates three distinct cellular pathways involved in inflammation and immunity, offering a more comprehensive assessment of the body’s inflammatory state (7, 10).

Evidence suggests that in older hypertensive patients, higher SIRI levels are associated with an increased cumulative incidence of total stroke, ischemic stroke, and hemorrhagic stroke. Elevated SIRI scores have been significantly linked to overall stroke risk and its subtypes, demonstrating superior predictive value compared to other inflammatory indices, such as the neutrophil-to-lymphocyte ratio (NLR), platelet-to-lymphocyte ratio (PLR), lymphocyte-to-monocyte ratio (LMR), and systemic immune-inflammatory index (SII) (11).

While the etiology and progression of ischemic stroke in young adults are closely linked to inflammation (12, 13), the specific relationship between the SIRI and young-onset ischemic stroke remains unclear. Furthermore, existing research has primarily focused on older populations, emphasizing the association between the SIRI levels and stroke risk (11). Therefore, this study aimed to investigate SIRI levels in young patients with acute ischemic stroke and explore their correlation with key clinical features, including infarct volume and distribution, intracranial artery stenosis, etiological classification, and types of vascular injury.

## Data and methods

### Research object

Patients aged 18 to 45 years with first-onset acute cerebral infarction who were hospitalized in the Department of Neurology at the Second Hospital of Hebei Medical University from 1 January 2022 to 31 October 2023 were selected as participants for the study. Additionally, non-acute cerebral infarction patients within the same age group and without intracranial atherosclerosis, who were hospitalized during the same period, were selected as control subjects.

The inclusion criteria were as follows: (i) age between18 and 45 years, with no restriction on sex; (ii) patients with first-onset acute cerebral infarction within 7 days of symptom onset, meeting the diagnostic criteria of the Chinese Guidelines for the Diagnosis and Treatment of Acute Ischemic Stroke 2018 and presenting neurological localization signs confirmed by cranial CT or cranial MRI examination.

**Figure 1:**
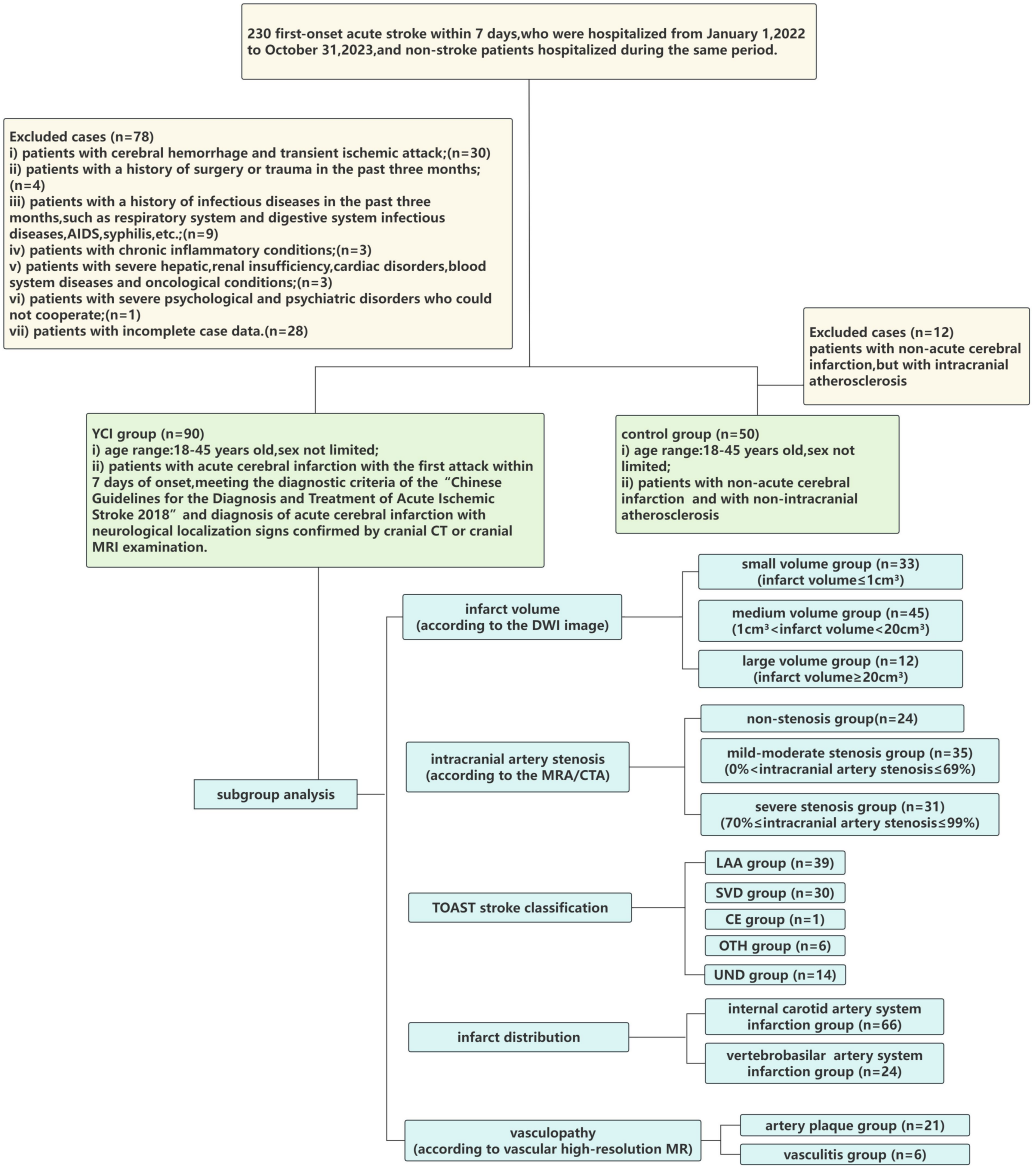
Flowchart of the study cohort.

The exclusion criteria were as follows: (i) patients with cerebral hemorrhage or transient ischemic attack; (ii) patients with a history of surgery or trauma within the past 3 months; (iii) patients with a history of infectious diseases within the past 3 months, such as respiratory and digestive system infections, AIDS, and syphilis; (iv) patients with chronic inflammatory conditions; (v) patients with severe hepatic, renal insufficiency, cardiac disorders, blood system diseases, or oncological conditions; (vi) patients with severe psychological and psychiatric disorders who could not cooperate; and (vii) patients with incomplete case data. As shown in Figure 1.

This retrospective cohort study was approved by the Clinical Research Ethics Committee of the Second Hospital of Hebei Medical University (registration number 2024-R663). The data were anonymized; therefore, informed consent was not required. This study was conducted in accordance with the Declaration of Helsinki. The study adhered to the Strengthening the Reporting of Observational Studies in Epidemiology (STROBE) guidelines to present its findings.

### Clinical data collection

General patient data, such as name, sex, age, body mass index (BMI), smoking and drinking history, and risk factors related to cerebral infarction (hypertension, diabetes, hyperlipidemia, immune system disease, and sleep disturbances), and imaging data (infarct volume, degree of intracranial artery stenosis, etiological classification, lesion distribution, and vascular high-resolution MR results), were collected.

On the second day after admission, laboratory indicators were collected from the patients, including homocysteine (HCY), uric acid (UA), creatinine (Cr), SIRI, and blood lipid levels (total cholesterol (TC), triglycerides (TG), high-density lipoprotein cholesterol (HDL-C), low-density lipoprotein cholesterol (LDL-C), and lipoprotein a (Lp-a)). The above blood indicators were measured by the Laboratory Department of the Second Hospital of Hebei Medical University using fully automated blood cell analyzers and biochemical analyzers.

### Grouping and observation projects

The research participants were divided into the youth cerebral infarction (YCI) group and the control group.

*YCI group*: A total of 90 hospitalized patients who met the inclusion criteria and did not meet any exclusion criteria were included.

*Control group*: A total of 50 patients of the same age group, hospitalized during the same period, who were confirmed to have no acute cerebral infarction by cranial CT or cranial MRI, had no intracranial arteriosclerosis as confirmed by MRA/CTA, and met none of the exclusion criteria, were included.

Further subgroup analysis was performed on the YCI group. The patients were divided into several subgroups according to infarct volume, intracranial artery stenosis, etiological classification, infarct distribution, and vasculopathy. The subgroup classifications included the following: (i) small-volume group (infarct volume≤1cm^3^), medium-volume group (1cm^3^ < infarct volume<20 cm^3^), and large-volume group (infarct volume≥20cm^3^) (according to the DWI image, infarct volume was calculated on DWI sequences by a trained radiologist who manually outlined the hyperintense infarct area on each slice. The total infarct volume was then computed by summing the areas across all slices and multiplying by the slice thickness); (ii) the non-stenosis group, the mild-to-moderate stenosis group (0% < intracranial artery stenosis≤69%), and the severe stenosis group (70% ≤ intracranial artery stenosis≤99%) (according to the MRA/CTA/Vascular high-resolution MR results); (iii) the large-vessel atherothromboembolic (LAA) group, cardioembolic (CE) group, small-vessel disease (SVD) group, stroke of other determined etiology (OTH) group, and stroke of undetermined etiology (UND) group (according to the Trial of ORG 10172 in Acute Stroke Treatment (TOAST) classification); (iv) the internal carotid artery system infarction group and the vertebrobasilar artery system infarction group; and (v) the artery plaque group and vasculitis group (according to the vascular high-resolution MR result).

The baseline characteristics of the YCI group and the control group were analyzed and compared. We used binary logistic regression to evaluate whether the SIRI is independently associated with YCI, and a receiver operating characteristic (ROC) curve was constructed. Additionally, the SIRI values were compared across the different subgroups of YCI patients.

### Statistical analysis

Statistical analysis was conducted using SPSS 29.0 software. Continuous variables that conformed to a normal distribution were expressed as mean ± standard deviation. A *t*-test was used to compare intergroup differences, and analysis of variance was used to compare differences among multiple groups. Continuous variables that did not conform to a normal distribution were expressed as median (interquartile range). The Mann–Whitney U-test was used to compare intergroup differences, while the Kruskal–Wallis test was used to compare differences among multiple groups. Categorical variables were expressed as frequency (percentage). A chi-squared test or Fisher’s exact test was used to compare intergroup differences. Binary logistic regression analysis was used to determine the factors associated with acute ischemic stroke in youth. For the logistic regression model, the Hosmer–Lemeshow test was used to assess the goodness of fit of the model, and a *p*-value of >0.05 indicated a good model fit. The variance inflation factor (VIF) was used to evaluate multicollinearity among independent variables, and a VIF value of <5 was considered to indicate the absence of serious multicollinearity. The ROC curve was used to evaluate the discriminative capacity of the SIRI for YCI. The optimal test cutoff point was determined by calculating Youden’s index. To evaluate the incremental discriminative value of the SIRI beyond conventional risk factors (age, smoking, hypertension, and HCY), logistic regression models with and without the SIRI were compared using the integrated discrimination improvement (IDI) and net reclassification improvement (NRI). All comparisons were two-tailed, and a *p*-value of < 0.05 was considered statistically significant.

### Sample size consideration

As a retrospective case–control study, all eligible patients available during the study period were included, resulting in a sample size of 90 patients in the youth cerebral infarction (YCI) group and 50 in the control group. A *post hoc* power analysis for the main effect was performed using G*Power version 3.1, applying a two-tailed Fisher’s exact test. The analysis revealed an observed statistical power of 90.41% for the association between the SIRI and YCI. In the subgroup analysis of the YCI patients with severe intracranial arterial stenosis, the observed statistical power for the association with the SIRI was 98.53%.

## Results

### Basic information of the YCI group versus the control group and the results of multivariable logistic regression analysis

Compared to the control group, the YCI group had statistically significant differences in smoking history and hypertension history (*p* = 0.008 and *p* = 0.002, respectively), whereas there were no significant differences between the two groups regarding age, sex, BMI, drinking history, diabetes history, hyperlipidemia history, immune system disease history, and sleep disturbances (*p =* 0.845, *p* = 0.475, *p* = 0.62, *p* = 0.729, *p* = 0.113, *p* = 0.841, *p* = 0.418, and *p* = 1.000, respectively). HCY and SIRI values were higher (*p <* 0.001 and *p* = 0.005, respectively), whereas HDL-C values were lower in the YCI group compared to the control group (*p* = 0.009). The data are shown in Table 1.

**Table 1:** Comparison of baseline characteristics and laboratory parameters between the YCI group and the control group

Parameters	YCI group (*n* = 90)	Control group (*n* = 50)	t/z/x^2^ value	*p* value
Age (years)	38.5 (34.75–41)	38 (33–43)	−0.196	0.845
Sex (Male/Female)	73/13	38/12	0.511	0.475
Smoking history (*n* %)	42 (46.7)	12 (24)	6.97	0.008*
Drinking history (*n* %)	24 (26.7)	12 (24)	0.12	0.729
Hypertension (*n* %)	44 (48.9)	11 (22)	9.74	0.002*
Diabetes (*n* %)	16 (17.8)	4 (8)	2.51	0.113
Hyperlipidemia (*n* %)	15 (16.7)	9 (18)	0.04	0.841
Immune system disease (*n* %)	3 (3.3)	4 (8)	0.655	0.418
Sleep disturbances (*n* %)	1 (1.1)	1 (2)	0.000	1.000
HCY	15.71 (11.47–34.32)	11.04 (8.72–13.03)	–4.895	<0.001*
Cr	72.22 ± 13.74	73.39 ± 11.61	–0.512	0.61
UA	383.64 ± 110.02	356.2 ± 99.33	1.463	0.15
SIRI	1.27 (0.85–2.07)	0.84 (0.61–1.5)	–2.794	0.005*
TC	4.51 ± 1.01	4.76 ± 0.84	–1.477	0.142
TG	1.52 (1.16–2.66)	1.35 (0.94–2.29)	–1.518	0.129
HDL-C	1.04 ± 0.23	1.15 ± 0.24	–2.656	0.009*
LDL-C	2.92 ± 0.93	3.13 ± 0.74	–1.406	0.162
Lp-a	10.31 (5.51–28.31)	10.44 (3.23–21.6)	–0.909	0.363
BMI	26.67 ± 4.02	26.32 ± 3.91	0.497	0.62

Data are presented as mean ± SD or median (Q1–Q3) for continuous variables and *n* (%) for categorical variables. *p<0.05 has statistical significance. HCY, homocysteine; Cr, creatinine; UA, uric acid; SIRI, systemic inflammatory response index; TC, cholesterol; TG, triglycerides; HDL-C, high-density lipoprotein cholesterol; LDL-C, low-density lipoprotein cholesterol; Lp-a, lipoprotein a; BMI, body mass index.

In the comparison between the YCI group and the control group, a *p*-value of <0.05 was analyzed as a significant univariable. After adjusting for potential confounders (smoking, hypertension, homocysteine, and HDL-C), the multivariate logistic regression model demonstrated that smoking (*p* = 0.043), hypertension (*p* = 0.028), homocysteine (*p* = 0.006), and SIRI (*p* = 0.032) were independently associated with cerebral infarction in young adults. The results are presented in Table 2.

**Table 2:** Multivariable logistic regression analysis of SIRI as a predictor of YCI

**Index**	**B value**	**Standard error**	**Wald Chi square value**	***p* value**	**OR** **(95%CI value)**
SIRI	0.526	0.246	4.582	0.032*	1.692 (1.045-2.739)
HDL-C	1.821	0.943	3.731	0.053	0.162 (0.026-1.027)
HCY	0.064	0.023	7.510	0.006*	1.066 (1.018-1.116)
Smoking History	0.900	0.445	4.086	0.043*	2.459 (1.028-5.884)
Hypertension	0.975	0.445	4.802	0.028*	2.652 (1.109-6.343)

Data are presented as mean±SD or median(Q1–Q3) for continuous variables and n(%) for categorical variables. *p<0.05 has statistical significance. SIRI, systemic inflammatory response index; HDL-C, high-density lipoprotein cholesterol; HCY, homocysteine.

The Hosmer–Lemeshow test indicated that the model fits well (*p* = 0.803). Collinearity diagnostics indicated that the model does not have serious multicollinearity issues (the VIF of all independent variables was <5).

**Figure 2:**
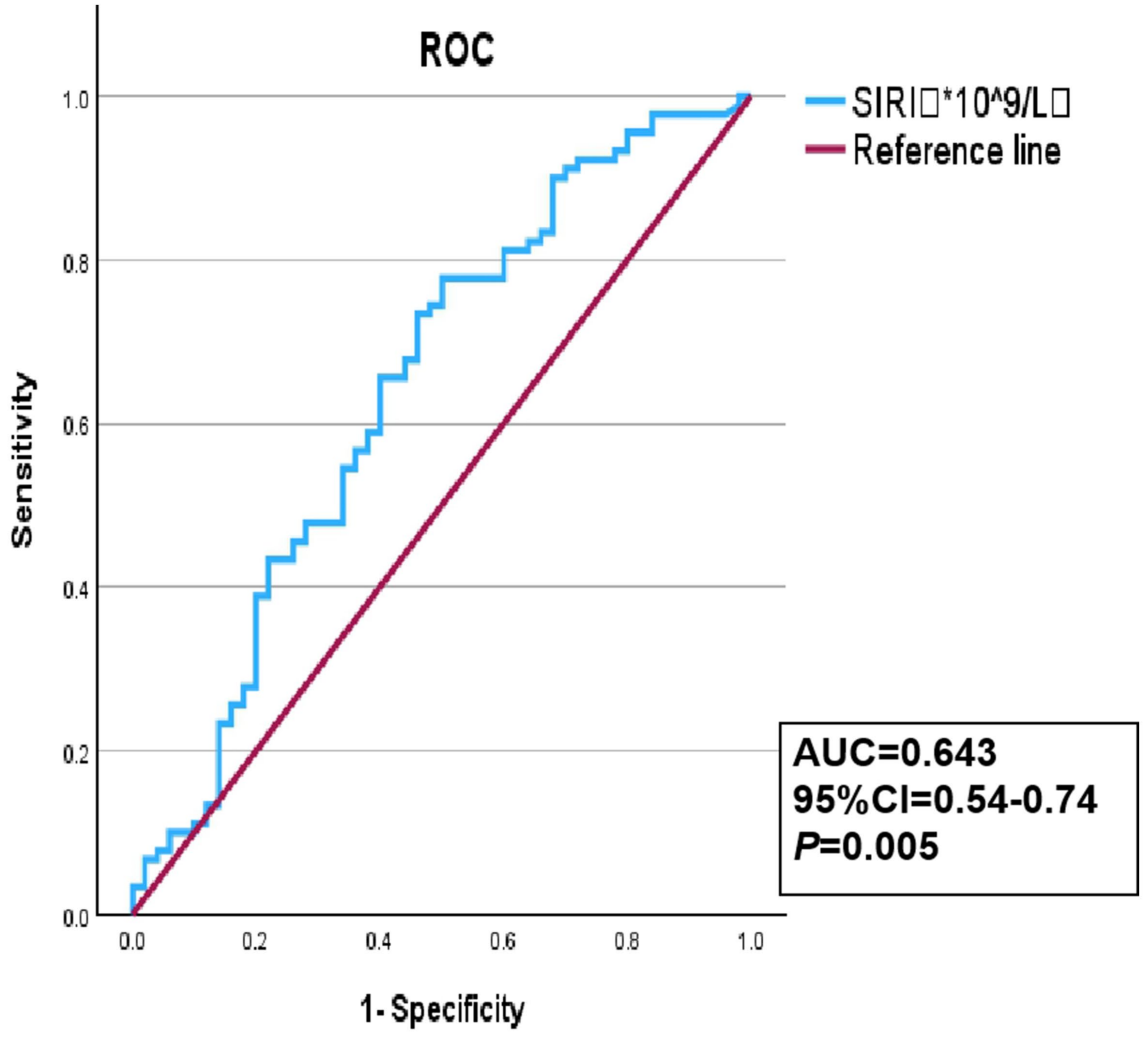
Receiver operating characteristic (ROC) curve of the SIRI discriminative ability for YCI.

The ROC curve was used to evaluate the discriminative capacity of the SIRI for YCI. The results showed that the optimal cutoff value of the SIRI as a discriminative indicator for YCI was 0.83*10^9/L, with a corresponding sensitivity and specificity of 77.8 and 50%, respectively. The AUC was 0.643, with a 95%CI of 0.54–0.74 and a *p*-value of 0.005, as shown in Figure 2.

To evaluate whether the SIRI adds incremental discriminative value beyond established clinical risk factors (age, smoking, hypertension, and HCY), we constructed a baseline clinical model. The AUC of the clinical model was 0.752 (95% CI:0.67–0.83). After adding the SIRI, the AUC increased to 0.768 (95% CI:0.69–0.85). The integrated discrimination improvement (IDI) was 0.021 (*p* = 0.042), and the net reclassification improvement (NRI) was 0.182 (*p* = 0.038), indicating a modest but significant improvement in reclassification and discrimination, as shown in Table 3.

**Table 3:** Improvement in predictive performance with addition of SIRI to traditional risk factors.

Metrics	Traditional model (HCY+Smoke+Hypertension)	Traditional model + SIRI (HCY+Smoke+Hypertension+SIRI)	Improvement	*P* value
Index (95% CI)	0.712	0.756	0.044 (0.005–0.083)	0.029*
NRI (95% CI)	–	–	0.324 (0.112–0.536)	0.003*
IDI (95% CI)	–	–	0.048 (0.016–0.080)	0.003*

Data are presented as mean ± SD or median(Q1-Q3) for continuous variables and *n* (%) for categorical variables. *p < 0.05 has statistical significance.

### Correlation analysis between SIRI values and infarct volume in YCI patients

**Figure 3:**
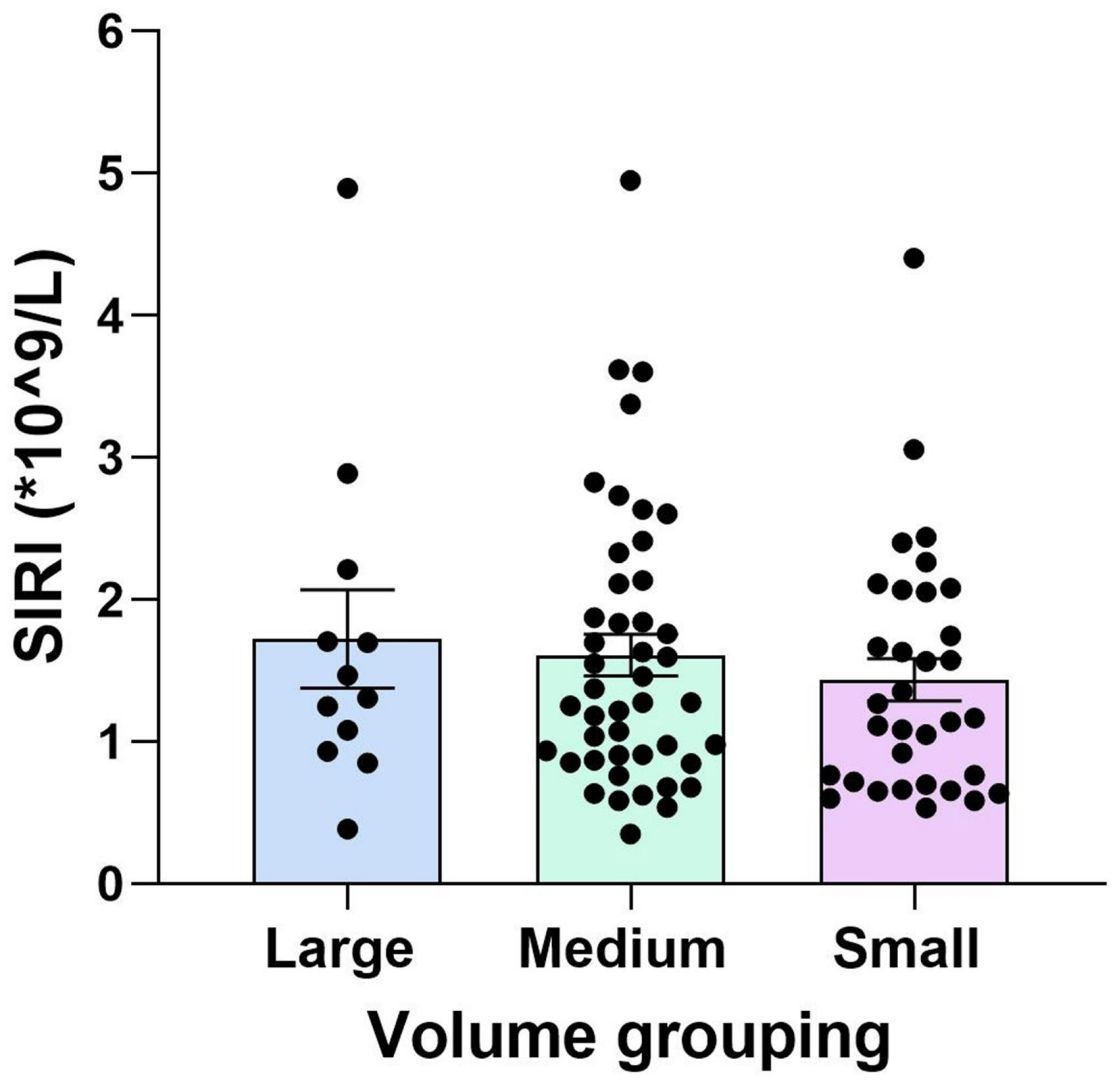
Comparison of the SIRI values among the large-, medium-, and small-volume groups in YCI. There was no statistically significant difference in the SIRI values among all the different volume groups (*p* = 0.633).

Further analysis was performed on the YCI patients. The patients were divided into subgroups based on infarct volume: the large-volume group (12 patients, 13.3%), the medium-volume group (45 patients, 50%), and the small-volume group (33 patients, 36.7%). The SIRI values for the large-volume group were higher than those for the medium-volume group, and the SIRI values for the medium-volume group were higher than those for the small-volume group. However, there was no statistically significant difference between the three groups (*p* = 0.633), as shown in Figure 3.

### Correlation analysis between SIRI values and intracranial arterial stenosis in YCI patients

**Figure 4:**
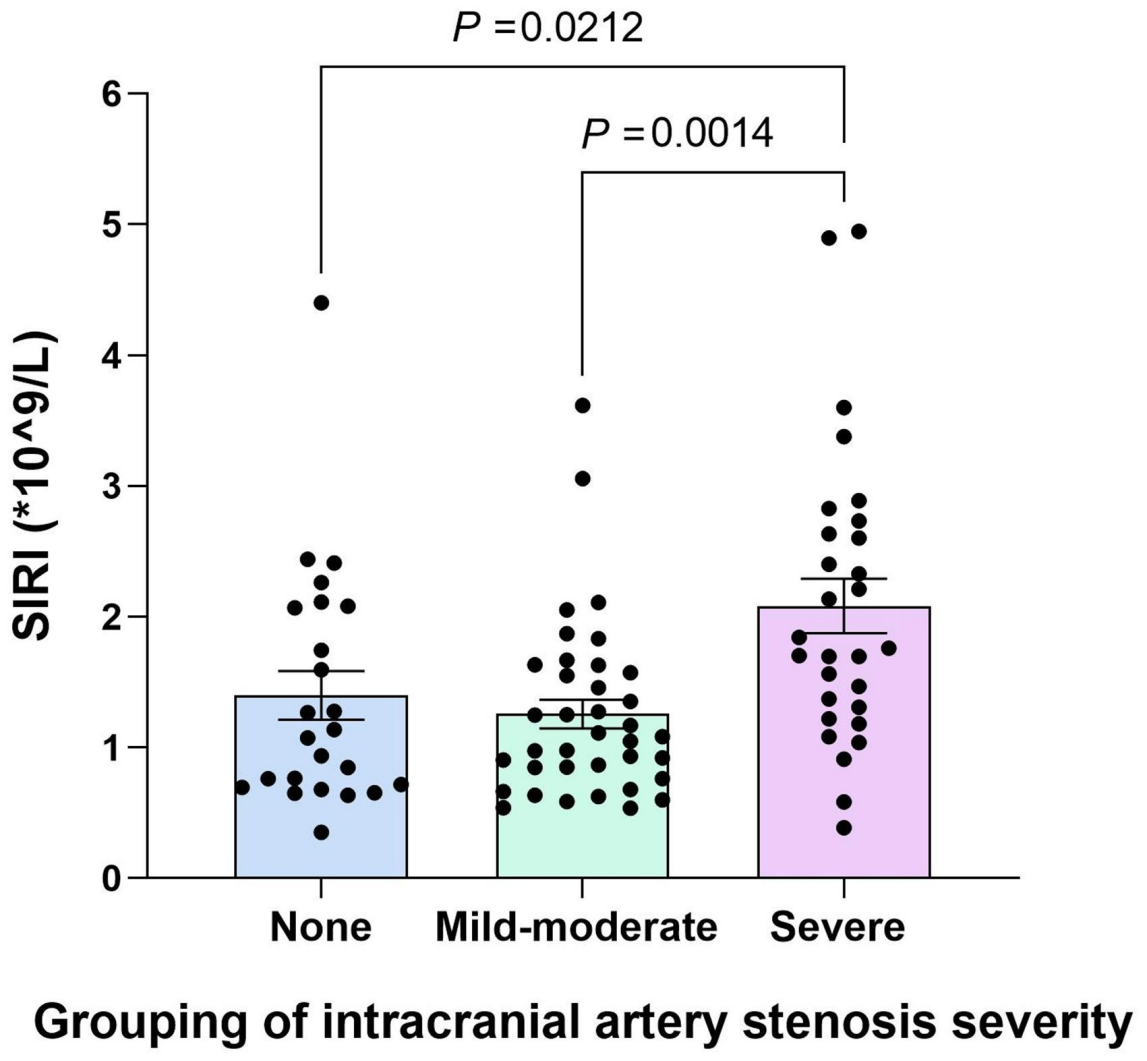
Comparison of the SIRI values among the non-stenosis group, the mild-to-moderate stenosis group, and the severe stenosis group. The SIRI values in the severe stenosis group were higher than those in the non-stenosis and mild–moderate stenosis groups (*p* < 0.001), and there was no statistically significant difference between the non-stenosis group and the mild–moderate stenosis group (*p* = 0.385).

The subgroups of the YCI patients according to intracranial arterial stenosis were as follows: the non-stenosis group (24 patients, 26.7%), the mild-to-moderate stenosis group (35 patients, 38.9%), and the severe stenosis group (31 patients, 34.4%). The SIRI values in the severe stenosis group were higher than those in the non-stenosis and mild-to-moderate stenosis groups (*p* = 0.003 and *p* < 0.001), and there was no statistically significant difference between the non-stenosis group and the mild-to-moderate stenosis group (*p* = 0.385), as shown in Figure 4.

Further analysis was conducted to assess whether the SIRI was independently associated with severe stenosis. The non-stenosis group and the mild-to-moderate stenosis group were combined into a single group called the non-severe stenosis group and compared with the severe stenosis group. The SIRI values in the severe stenosis group were higher than those in the non-severe stenosis group (*p* < 0.001). After adjusting for confounding factors, such as age, sex, hypertension, diabetes, smoking, and HCY, multivariable logistic regression analysis revealed that the SIRI was independently associated with severe stenosis (original OR = 3.346, 95% CI = 1.761–6.359, *p* < 0.001; corrected OR = 5.278, 95% CI = 2.317–12.022, *p* < 0.001). The Hosmer–Lemeshow test indicated that the model fits well (*p* = 0.805). Collinearity diagnostics indicated that the model does not have serious multicollinearity issues (the VIF of all independent variables was <5), as shown in Table 4.

**Table 4:** Multivariable logistic regression analysis of SIRI as a risk factor for severe stenosis of intracranial arteries.

Index	Original OR (95% CI)	*p* value	Correction OR (95% CI)	*p* value
SIRI	3.346 (1.761–6.359)	<0.001*	5.278 (2.317–12.022)	<0.001*

*Data are presented as mean±SD or median(Q1-Q3) for continuous variables and n(%) for categorical variables. *p* < 0.05 indicates statistically significant differences.

SIRI, Systemic inflammatory response index.

### Correlation analysis between SIRI values and the TOAST classification in YCI patients

The YCI patients were divided into subgroups according to the TOAST classification. There were 39 patients (43.3%)in the LAA group, 30 patients (33.3%)in the SVD group,1 patient (1.1%) in the CE group,6 patients (6.7%) in the OTH group, and 14 patients (15.6%) in the UND group. The CE group, OTH group, and UND group were not statistically analyzed due to the small number of cases. The SIRI values in the LAA group were higher than those in the SVD group (*p* = 0.03), as shown in Table 5.

**Table 5:** Comparison of SIRI values and etiological classification

Group	*n*	SIRI (*10^9/L)	*p* value
LAA group	39	1.63(1.04-2.40)	0.03*
SVD group	30	1.12(0.69-1.82)	

Data are presented as mean±SD or median(Q1-Q3) for continuous variables and n(%) for categorical variables. *p < 0.05 has statistical significance. SIRI, systemic inflammatory response index.

### Correlation analysis between SIRI values and infarct distribution in YCI patients

The YCI patients were grouped based on infarct distribution in either the internal carotid artery system or the vertebrobasilar artery system. There were 66 patients (73.3%) in the internal carotid artery system infarction group and 24 patients (26.7%) in the vertebrobasilar artery system infarction group. There was no statistically significant difference in the SIRI values between the different infarct distribution groups (*p* = 0.572), as shown in Table 6.

**Table 6:** Comparison of SIRI values and lesion distribution.

Group	*n*	SIRI (*10^9/L)	*p* value
Internal carotid artery system infarction group	66	1.33 (0.90–1.92)	0.572
Vertebrobasilar artery system infarction group	24	1.15 (0.67–2.11)	

Data are presented as mean±SD or median(Q1-Q3) for continuous variables and *n* (%) for categorical variables. There is no statistically significant difference when *p* > 0.05. SIRI, Systemic inflammatory response index.

### Correlation analysis between SIRI values and vasculopathy in YCI

A total of 27 patients in the YCI group underwent vascular high-resolution MR, and the vascular damage types were used as the basis for subgrouping. There were 21 patients (77.8%) in the arterial plaque group and 6 patients (22.2%) in the vasculitis group. The SIRI levels in the arterial plaque group were higher than those in the vasculitis group. However, there was no statistically significant difference between the two groups (*p* = 0.345), as shown in Table 7.

**Table 7:** Comparison of SIRI values and vasculopathy.

Group	*n*	SIRI (*10^9/L)	*p* value
Arterial plaque group	21	1.25 (0.89–1.84)	0.345
Vasculitis group	6	0.92 (0.73–2.03)	

Data are presented as mean±SD or median(Q1-Q3) for continuous variables and *n* (%) for categorical variables. There is no statistically significant difference when *p* > 0.05. SIRI, Systemic inflammatory response index.

## Discussion

Inflammatory biomarkers have received increasing attention in recent years. As inflammatory indicators, the NLR, PLR, LMR, and red blood cell distribution width (RDW) have all been reported to predict stroke prognosis (14–16). The SIRI is an emerging inflammatory marker that is a more comprehensive chronic low-grade inflammation marker based on monocyte, neutrophil, and lymphocyte counts. It has been proven to effectively reflect the inflammatory state and predict the prognosis of many diseases, such as cancer and coronary artery disease (17–22). The majority of previous studies have shown that the SIRI is associated with the severity, prognosis, and related complications of ischemic stroke (23–25). Another study showed that the SIRI can predict the functional status of patients with critical acute ischemic stroke at discharge (26). In addition, studies have explored the relationship between dyslipidemia, PLR, and ischemic stroke in young adults (3, 4). However, research on the relationship between the SIRI and acute ischemic stroke in young people is still lacking. This study investigated the SIRI in YCI and its correlations with infarct volume, lesion distribution, intracranial arterial stenosis, etiological subtypes, and the nature of vascular pathology. Our findings revealed significantly elevated SIRI levels in these patients. Even after adjusting for confounding factors such as smoking, hypertension, and homocysteine levels, the SIRI remained an independent correlated factor of YCI, suggesting it is a significant associated factor. Furthermore, the SIRI demonstrated utility in distinguishing between the large-artery atherosclerosis and small-artery occlusion subtypes. A high SIRI level was also indicative of a greater likelihood of severe intracranial arterial stenosis.

The link between the SIRI and YCI may stem from both the acute-phase inflammation post-infarction and the chronic inflammation associated with conditions such as atherosclerosis. Therefore, the SIRI serves as an integrated measure of this total inflammatory burden.

The acute phase of cerebral infarction involves key pathological processes including blood–brain barrier disruption, oxidative stress, and inflammation (27). The inflammatory cascade, orchestrated by complex interactions among various cells, plays a pivotal role in influencing infarct progression, resolution, and subsequent tissue repair and remodeling (28). Cerebral infarction triggers the release of inflammatory mediators from injured brain tissue, leading to the systemic release of neutrophils/monocytes and lymphocyte apoptosis/dysfunction. The infiltrating myeloid cells initiate a neuroinflammatory cascade within the brain, increasing capillary permeability and promoting thrombosis. Meanwhile, the loss of lymphocyte function results in impaired immune defense and overall immune imbalance (29). The SIRI is a composite index derived from neutrophil, monocyte, and lymphocyte counts (SIRI = neutrophils*monocytes/lymphocytes) (10). This integrative nature of the immune response directly explains the elevated serum SIRI levels observed in the young patients with cerebral infarction.

The independent association between the SIRI and YCI indicates that the systemic inflammatory response, as revealed by the SIRI, constitutes an important component of the pathophysiology of this condition. The ROC curve analysis demonstrated that the SIRI possesses a certain discriminative ability to distinguish young individuals with acute cerebral infarction from those without infarction. Its relatively high sensitivity suggests that SIRI can effectively identify the majority of YCI patients, particularly those with an inflammatory component. Consequently, in clinical practice, an abnormally elevated SIRI value could be regarded as a “warning signal,” indicating the need for more comprehensive cerebrovascular assessment and inflammatory risk management. The moderate specificity of the SIRI in distinguishing YCI may be attributed to its nature as a non-specific systemic inflammatory marker. Elevated SIRI levels could result from mild, subclinical inflammatory conditions that are common among young adults, which are difficult to completely exclude despite strict study criteria. Furthermore, the etiology of stroke in young adults is highly heterogeneous; not all cases are strongly linked to systemic inflammation, which also affects the specificity of the SIRI.

However, after adding the SIRI to the traditional clinical model (including smoking, hypertension, and homocysteine), improvements in the C-index, NRI, and IDI were observed. This underscores the value of the SIRI as a discriminative indicator. The SIRI captures an independent aspect of disease pathophysiology, the inflammatory response, which cannot be fully reflected by homocysteine, smoking, or hypertension alone. Future prospective, longitudinal studies that measure the SIRI in healthy populations and track their stroke outcomes are warranted to clarify its potential as a true predictive risk factor.

Chronic systemic inflammation mediates the development of atherosclerosis, which is itself a major cause of cerebral infarction (30, 31).In the progression of atherosclerosis, neutrophils amplify inflammatory responses and induce endothelial injury through the release of reactive oxygen species and neutrophil extracellular traps; monocytes, as precursors of macrophages, infiltrate the vascular wall and engulf lipids to form foam cells, constituting the core of atherosclerotic plaques; and lymphocyte apoptosis impairs protective immunoregulatory capacity, thereby exacerbating inflammatory damage (32). Studies have shown that patients with carotid plaques exhibit higher serum SIRI levels, indicating that the SIRI holds predictive or indirect assessment value for atherosclerosis (31). This conclusion is consistent with the findings of the present study. Therefore, the SIRI reflects the inflammatory burden that promotes atherosclerosis. This mechanism explains why YCI patients with the large-artery atherosclerosis subtype exhibited higher SIRI levels and why the SIRI emerged as an independent correlate of severe intracranial arterial stenosis. The clinical relevance of our finding lies in the close association between the SIRI and a specific subtype of YCI, which may help identify patients with more intense inflammation and a greater atherosclerotic burden. In practice, the SIRI could assist clinicians in rapidly recognizing patients who might benefit from aggressive anti-inflammatory and lipid-lowering therapy.

However, it should be noted that the comparison between LAA and SVD was conducted in a subset of participants, in which the CE, OTH, and UND subtypes were not statistically analyzed due to the small number of cases. Therefore, although the finding is statistically significant and biologically plausible, it should be regarded as exploratory and warrants further validation in larger cohorts that encompass all etiological subtypes.

Currently, there are no reports on the correlation between the SIRI and infarct volume/distribution/vascular damage type. We found that the SIRI showed no statistically significant differences with cerebral infarction volume, anterior and posterior circulation infarction distribution, or artery plaques and vasculitis. The reasons may be as follows: (i) The SIRI reflects the inflammatory status of the body but has no specificity for certain types of vessel damage. (ii) Due to the small sample size of the infarct volume group and the vascular lesion group, the lack of statistical difference in the SIRI values between these two subgroups cannot be interpreted as having no association; it may instead reflect insufficient sample size to detect an association. Therefore, it should not be regarded as a definitive negative result or no biological association. The original intention behind conducting this analysis was to utilize these valuable data for a preliminary exploratory study, representing initial results. Future research should expand the sample size for verification. Therefore, further studies are needed to improve and explore the SIRI in YCI.

### Limitations and future directions

This study has some limitations. First, although multifactorial adjustments were made, there were still unmeasurable confounders, such as diet, genetics, socioeconomic status, and the use of anti-inflammatory medications. Future research employing methods less susceptible to confounding—such as Mendelian randomization studies to assess causality or prospective cohorts designed to comprehensively collect data on complex lifestyle and environmental factors—is crucial to validate and extend our findings. Second, only the baseline level of the SIRI was calculated, and the impact of dynamic changes in the SIRI on YCI was not observed. Therefore, it is unclear whether the variation in SIRI over time will affect its value, warranting further study in the future. Third, this study was a single-center retrospective study. Although we implemented strict standards and rigorous statistical adjustments, the generalizability of our findings may be limited by selection bias and the characteristics of the local patient population. These findings should be validated using large, multicenter prospective cohorts to ensure their reliability and applicability. Finally, while our sample size demonstrates sufficient statistical power for the primary association, it remains relatively limited, particularly for the subgroup analyses (such as volumetric groups and vasculopathy types). The negative findings in these subgroups should be interpreted with caution, as they may stem from limited statistical power rather than a true absence of association, and are best considered exploratory. Larger-scale longitudinal studies are needed to investigate the predictive value of the SIRI in stroke risk assessment over time.

## Conclusion

Our retrospective study demonstrated that an elevated SIRI level is an independent correlate of acute ischemic stroke in young adults. Strikingly, the SIRI was specifically linked to severe intracranial arterial stenosis and the large-artery atherosclerotic subtype, indicating its clinical utility in pinpointing patients with more pronounced inflammatory atherosclerosis. These findings underscore the potential of the SIRI and emphasize the need for confirmation through future large-scale prospective studies.

## Data Availability

The raw data supporting the conclusions of this article will be made available by the authors, without undue reservation.
